# Management of a complex transjugular intrahepatic portosystemic shunt dysfunction with endotipsitis through rotational thrombectomy

**DOI:** 10.1093/bjrcr/uaae005

**Published:** 2024-02-08

**Authors:** Francesco Vizzutti, Emanuele Casamassima, Gianmarco Falcone, Giacomo Gabbani, Martina Rosi, Valentina Adotti, Fabio Marra, Fabrizio Fanelli

**Affiliations:** Department of Experimental and Clinical Medicine, University of Florence, Florence I-50134, Italy; Interventional Radiology Unit, Department of Radiology, Careggi University Hospital, Florence I-50134, Italy; Interventional Radiology Unit, Department of Radiology, Careggi University Hospital, Florence I-50134, Italy; Interventional Radiology Unit, Department of Radiology, Careggi University Hospital, Florence I-50134, Italy; Department of Experimental and Clinical Medicine, University of Florence, Florence I-50134, Italy; Department of Experimental and Clinical Medicine, University of Florence, Florence I-50134, Italy; Department of Experimental and Clinical Medicine, University of Florence, Florence I-50134, Italy; Center for Research, High Education and Transfer DENOThe, University of Florence, Florence I-50134, Italy; Interventional Radiology Unit, Department of Radiology, Careggi University Hospital, Florence I-50134, Italy

**Keywords:** TIPS, portal vein thrombosis, endotipsitis, biliary fistula, rotational thrombectomy, EHPVO

## Abstract

Transjugular intrahepatic portosystemic shunting (TIPS) is an established strategy for the management of complications of portal hypertension. Endoprosthetic infection (“endotipsitis”) is a rare but serious and difficult-to-treat complication of TIPS placement. Here we report the occurrence of an infected thrombus complicating TIPS placement in a patient with extra-hepatic portal vein obstruction, recurrent variceal bleeding and portal biliopathy accompanied by recurrent cholangitis. Infected thrombotic material within TIPS could be removed only by employing rotational thrombectomy. This procedure revealed the presence of a biliary fistula which carried pathogens in the systemic circulation. The multiple episodes of sepsis did no longer recur following exclusion of the biliary fistula. This case highlights the possibility to use rotational thrombectomy for the management of complex cases of TIPS dysfunction.

## Case presentation

In patients with chronic extra-hepatic portal vein obstruction (EHPVO), portal vein (PV) recanalization with or without trans-jugular intra-hepatic portosystemic shunt (TIPS) can be considered in the presence of portal hypertension-related complications.^1^ In this clinical setting, the TIPS procedure may be challenging and possibly associated with a higher burden of procedural complications, whose management could require innovative approaches.

**Figure 1.**
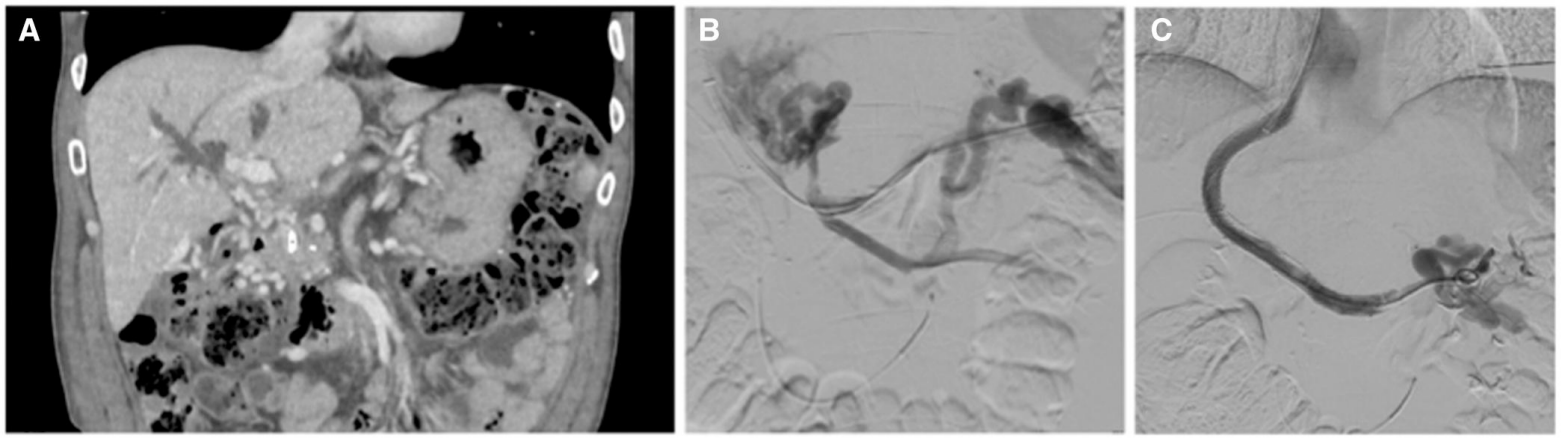
(A) Contrast enhanced CT scan, venous phase, showing cavernomatous transformation of extra-hepatic portal vein and portal biliopathy. (B) Catheterization of the native portal vein by a combined percutaneous trans-jugular intra-hepatic and trans-splenic approach. (C) Final phlebography at TIPS positioning.

A 60-years old Caucasian man with PV thrombosis complicating chronic alcoholic pancreatitis was scheduled for TIPS placement as secondary prophylaxis of variceal bleeding^2^ (Figure 1A). The clinical picture was also complicated by portal biliopathy and recurrent cholangitis,^3^ secondary to the development of a PV cavernoma. There was no evidence of underlying liver disease and parameters of liver function were normal except for mild hyperbilirubinemia (2.8 mg/dL) with prevalence of the conjugated fraction. We first performed percutaneous trans-hepatic cholangiography and to resolve biliary tree dilatation caused by the cavernoma, an 8.3 Fr internal-external drainage catheter (Ring biliary catheter, Cook, Bloomington, IN, United States) was positioned. The patient was then scheduled for a TIPS procedure. Under ultrasound guidance, a landing intra-hepatic PV branch was reached by percutaneous trans-jugular intrahepatic approach, and the native PV trunk was catheterized by combining a trans-splenic approach to assist with the recanalization of the portal venous system (Figure 1B).^4^ A Viatorr CX 8 + 2 cm endoprosthesis (Gore, Flagstaff, AZ, United States) was deployed from the ostium of the right hepatic vein in the inferior vena cava to the confluence of native splenic (SV) and superior mesenteric veins (SMV). The portal-systemic derivation was completed by a Wallstent (Boston Scientific, Marlborough, MA, United States) coaxially deployed to reach the patent middle portion of the SV, thereby not hindering the systemic derivation of collateral vessels draining the SMV territory. The portacaval pressure gradient (PCG) dropped (12 vs. 27 mmHg) following 10 mm balloon dilation (Figure 1C).

Three days after the TIPS procedure the patient developed a septic shock with isolation of multiple organisms (*Pseudomonas aeruginosa, Enterococcus faecium, Hafnia alvei, Klebsiella oxytoca, Klebsiella pneumoniae, Streptococcus* spp., and *Candida albicans*) in blood cultures. Despite targeted antimicrobial treatment and recover of haemodynamic, renal, and neurologic function, septic fever and bacteraemia recurred, and the patient developed respiratory failure related to multifocal bilateral pneumonia, suggestive of haematogenic bacterial spread.

## Investigations/imaging findings

**Figure 2.**
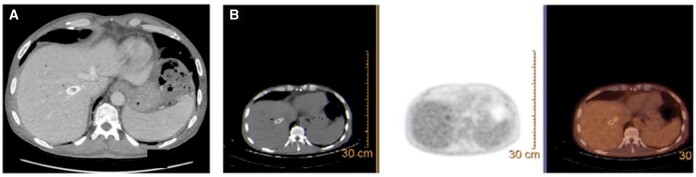
(A) Contrast enhanced CT scan, venous phase, showing thrombotic material within the intra-parenchymal portion of the endoprosthesis; (B) PET with 2-deoxy-2-[fluorine-18]fluoro-D-glucose showing FDG avid uptake within the thrombosed TIPS.

After a complete clinical workout, contrast-enhanced ultrasonography and CT scan (Figure 2A) demonstrated a TIPS dysfunction, with sub-occluding thrombosis located in the intrahepatic portion. A PET with 2-deoxy-2-[fluorine-18]fluoro-D-glucose integrated with CT was compatible with endotipsitis (Figure 2B). Angiographic revision confirmed thrombotic sub-occlusion of TIPS with an elevated PCG (24 mm Hg). Using endoscopic forceps (CITEC, Changzhou Jiangsu, China) advanced through an introducer placed within the covered portion of TIPS, a specimen of the occluding thrombus was obtained for culture, and tested positive for *C. albicans* and *E. faecium*, definitively confirming endotipsitis.

## Treatments

**Figure 3.**
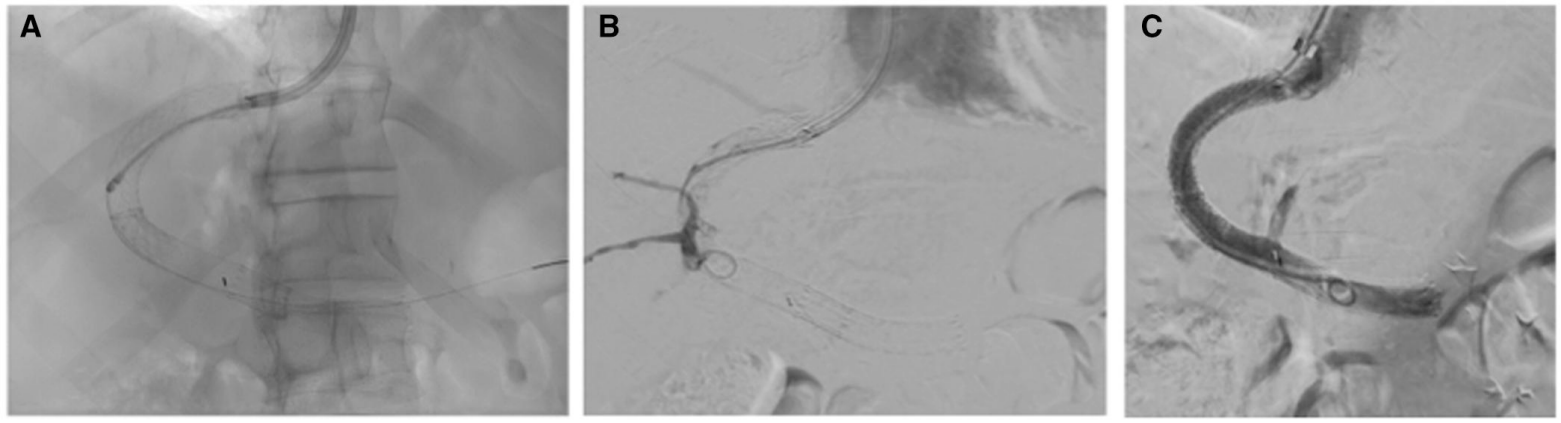
(A) Use of a rotational thrombectomy to treat the persistent TIPS thrombosis; (B) Catheterization of a biliary fistula identified following TIPS re-habitation; (C) exclusion of the biliary fistula following co-axial distal deployment of an additional covered endoprosthesis.

Using mechanical thrombectomy (Angiojet, Boston Scientific, Marlborough, MA, United States) with 50 mg recombinant tissue plasminogen activator and 10 mm angioplasty, TIPS patency was only partially recovered due to persistence of organized and stiff thrombotic material in the intra-parenchymal portion of the endoprosthesis, occluding approximately 40% of the lumen. Thrombotic sub-occlusion of TIPS rapidly recurred and fever and bacteraemia persisted despite targeted antimicrobial treatment. One week later, a further revision was scheduled and a device for rotational thrombectomy (Aspirex system, Straub Medical, Wangs, Switzerland) was engaged within the covered portion of TIPS, directing it through a bi-directional steerable guiding sheath (Destino twist, Oscor, Palm Harbor, FL, United States). This procedure resulted in a near-complete recanalization of the TIPS (Figure 3A) at control angiography and endovascular ultrasonography, with approximately 10 mm long thrombotic apposition occluding less than 10% of the lumen, within the intrahepatic portion of endoprosthesis. Notably, control angiography revealed a biliary fistula located at the passage between the covered and uncovered portion of the endoprostheses, communicating with the right intra-hepatic biliary system (Figure 3B). Therefore, an Advanta 10-mm endoprosthesis (Getinge, Merrimack, NH, United States) was released to close the fistula (Figure 3C). The PCG dropped to 13 mm Hg. The patient was maintained on targeted antibiotic and antifungal treatment for four weeks following hospital discharge.

## Outcome and follow-up

At the 9-month follow-up, no further TIPS dysfunction, bacteraemia, fever, or sepsis had occurred. No recurrence of complications of portal hypertension or portal biliopathy were recorded and the patient was kept on oral anticoagulant (warfarin).

## Discussion

Endotipsitis is a serious complication, with a high mortality burden (approximately 30%).^5^^,^^6^ Only a few cases of endotipsitis have been described in the literature, and standard and effective management strategies are lacking. Definitive treatment often requires liver transplantation, which is not always feasible. Moreover, the wider use of TIPS may lead to increased incidence of this difficult-to-treat complication, especially in technically complex cases such as cavernomatous transformation of the PV. Currently, the treatment of endotipsitis relies on broad-spectrum antibiotics possibly targeted based on pathogen isolation in cultures.

In the case presented herein a fistulous communication with the biliary system, not highlighted at TIPS positioning, was hidden by a stiff thrombus adhered to the endoprosthesis. Notably, the patient never presented haemophilia or jaundice, probably in relation to the very small size of the communication. On the other hand, the fistula led to infection of the thrombus and allowed the systemic passage of bacteria and fungi from the biliary tree, chronically colonized with a polymicrobial flora. Moreover, the contribution of further colonization of the biliary tree following internal-external drainage catheter placement could not be excluded. As a result, antibiotic treatment controlled the septic state, but persistence of the fistulous communication prevented the resolution of endotipsitis and spreading of micro-organisms to the systemic circulation.

It is important to note that approaches conventionally used for the management of TIPS thrombosis were unsuccessful in this case, probably due to the physical characteristics of the thrombus. Only the use of rotational thrombectomy engaged within the covered portion of the endoprosthesis permitted sufficient fragmentation of organized thrombotic material, allowing the demonstration of the biliary fistula. The pathogenetic role of the fistula is clearly demonstrated by the fact that episodes of sepsis did not recur after exclusion of the biliary system from the systemic circulation.

## Learning points

- This report describes for the first time the successful use of rotational thrombectomy for the management of endotipsitis in a case of infected thrombosis.
- Importantly, care must also be taken in the manipulation of the bile ducts in potential candidates to TIPS postponing it, when possible, to the resolution of the biliary obstruction.
